# Heme oxygenase and the immune system in normal and pathological pregnancies

**DOI:** 10.3389/fphar.2015.00084

**Published:** 2015-04-24

**Authors:** Maide Ozen, Hui Zhao, David B. Lewis, Ronald J. Wong, David K. Stevenson

**Affiliations:** ^1^Division of Neonatal and Developmental Medicine, Department of Pediatrics, Stanford University School of Medicine, Stanford, CA, USA; ^2^Division of Allergy, Immunology, and Rheumatology, Department of Pediatrics, Stanford University School of Medicine, Stanford, CA, USA

**Keywords:** fetus, HO-1, immunomodulation, immunotolerance, newborn, placenta, pregnancy

## Abstract

Normal pregnancy is an immunotolerant state. Many factors, including environmental, socioeconomic, genetic, and immunologic changes by infection and/or other causes of inflammation, may contribute to inter-individual differences resulting in a normal or pathologic pregnancy. In particular, imbalances in the immune system can cause many pregnancy-related diseases, such as infertility, abortions, pre-eclampsia, and preterm labor, which result in maternal/fetal death, prematurity, or small-for-gestational age newborns. New findings imply that myeloid regulatory cells and regulatory T cells (Tregs) may mediate immunotolerance during normal pregnancy. Effector T cells (Teffs) have, in contrast, been implicated to cause adverse pregnancy outcomes. Furthermore, feto-maternal tolerance affects the developing fetus. It has been shown that the Treg/Teff balance affects litter size and adoptive transfer of pregnancy-induced Tregs can prevent fetal rejection in the mouse. Heme oxygenase-1 (HO-1) has a protective role in many conditions through its anti-inflammatory, anti-apoptotic, antioxidative, and anti-proliferative actions. HO-1 is highly expressed in the placenta and plays a role in angiogenesis and placental vascular development and in regulating vascular tone in pregnancy. In addition, HO-1 is a major regulator of immune homeostasis by mediating crosstalk between innate and adaptive immune systems. Moreover, HO-1 can inhibit inflammation-induced phenotypic maturation of immune effector cells and pro-inflammatory cytokine secretion and promote anti-inflammatory cytokine production. HO-1 may also be associated with T-cell activation and can limit immune-based tissue injury by promoting Treg suppression of effector responses. Thus, HO-1 and its byproducts may protect against pregnancy complications by its immunomodulatory effects, and the regulation of HO-1 or its downstream effects has the potential to prevent or treat pregnancy complications and prematurity.

## Introduction

There are many physiological adaptations that are essential for a healthy pregnancy. Profound immunological changes must take place such as dynamic alterations in the proportions of immune cells in the maternal blood and decidua and of highly specific decidual immune cell types that arise only during pregnancy. These changes allow for the host to become “non-reactive” to the allogeneic fetus and for the establishment of an immunotolerant environment for this “allogeneic graft” so that a successful implantation can occur and a healthy, but permissive, feto-placental barrier is maintained. This complex adaptive process can be disrupted by maternal and fetal inflammation or infections. It has been shown that both epigenetic (environmental factors such as pollutants, nutrition, stress; Murphy, 2007; Shachar et al., 2013; Patel et al., 2014) and genetic factors (Kaartokallio et al., 2014) cannot only affect an ongoing pregnancy and subsequent outcomes and result in phenotypic alterations in the offspring; but also, can result in germ-line alterations that lead to adverse transgenerational effects (Skinner, 2014). Heme oxygenase (HO), a ubiquitous and genetically polymorphic enzyme, has three isozymes—the inducible HO-1, the constitutive HO-2, and HO-3, which appears to be a pseudogene (McCoubrey et al., 1997). HO is the rate-limiting step in the heme catabolic pathway, producing equimolar amounts of iron, carbon monoxide (CO), and biliverdin that is then reduced to bilirubin. HO-1 has protective effects in many disease states through its anti-inflammatory, anti-apoptotic, antioxidative, and anti-proliferative actions (Woo et al., 1998; Zenclussen et al., 2005). Because it also has immunosuppressive properties, HO-1 may also have a role in maintaining the immune balance in a normal pregnancy, as discussed below, is supported by the observations that suboptimal expression of HO-1 has been shown to be associated with pregnancy, fetal, and neonatal complications.

## Immune System and Immunotolerance in Normal and Pathologic Pregnancies

A normal pregnancy can be regarded as a series of mechanisms regulating immunotolerance, starting as early as ovulation and occurring primarily at the feto-placental junction. Based largely on murine studies, this traditionally has been attributed to a T helper 2 (Th2)-skewed immunity in which the production of IL-4, IL-5, and IL-13 by CD4 T cells is prominent. However, recent studies have shown that this process is much more complex, and that Th2 responses may not play a central role in feto-maternal tolerance in humans (Saito et al., 2010; Dimova et al., 2011; Liu et al., 2011; Schlossberger et al., 2013; Zhao et al., 2015). The immune cell phenotypes at the feto-placental junction are considerably different than their peripheral (circulating) counterparts (Saito et al., 2010; Arck and Hecher, 2013). In the establishment and maintenance of an immunotolerant feto-placental environment, the changing phenotypes of key immune cells may be essential. Many immune cell types undergo changes in phenotypes and proportions during normal pregnancy and at the time of parturition. This immunotolerant state is perturbed during pathological pregnancies.

### Contributors to Immunotolerance During Normal Pregnancy

#### Decidua/Placenta

Early in human pregnancies, a tolerogenic phenotype predominates in the decidua during implantation of the fetus, which because of its expression of major and minor histocompatibility antigens differing from that of the mother, can be considered an allograft. Uterine natural killer (uNK) cells (Zhao et al., 2011; Le Bouteiller, 2013) and M2 (alternative) macrophages help with the initial steps of this process by mediating uterine spiral artery remodeling, trophoblastic invasion, and immunomodulation (Nagamatsu and Schust, 2010b; Houser, 2012). For example, unlike circulating monocyte-derived macrophages after the uptake of apoptotic trophoblasts, placental M2 macrophages do not produce inflammatory mediators (Ben Amara et al., 2013), and hence may limit immune responses and promote immunotolerance. However, as parturition nears, placental macrophages become predominately an inflammatory (M1) type (Nagamatsu and Schust, 2010a), contributing to the “physiologic inflammation” associated with the initiation of parturition. Decidual stromal cells (DSCs) may also contribute to supporting a healthy feto-placental environment during the first trimester in healthy human pregnancies. For example, when human decidua cells of the first trimester of pregnancy were co-cultured with healthy unrelated donor lymphocytes, DSCs were found to inhibit NK-cell function, dendritic cell (DC) differentiation, and T-cell responses (Croxatto et al., 2014). In addition, high numbers of regulatory T cells (Tregs) at the feto-maternal interface present from early to mid-gestation (Guerin et al., 2009) have been shown to be associated with successful implantation in humans (Guerin et al., 2009) as well as in mice (Zenclussen et al., 2007). As pregnancy advances, uNK cells and Tregs decrease in the human and mouse deciduae. These changes are accompanied by changes in the composition of the Treg pool. For example, in pregnant mice, there is a predominance of thymic-derived (natural) nTregs that rapidly decrease and an increase of peripherally-induced Tregs (iTregs) in the blood and uterine lymph nodes by the conversion of peripheral naïve T cells to iTregs (Teles et al., 2013). In addition, the two classical Treg cell populations (CD4^+^CD25^++^FoxP3^+^ and CD4^+^CD25^+^FoxP3^+^) and putative naïve Tregs (CD4^+^CD25^–^FoxP3^+^) increase significantly in the decidua of healthy pregnant women (Dimova et al., 2011). These T-cell changes help sustain a healthy allograft/host environment.

#### Peripheral Blood

Although the total number of circulating Tregs are not different between pregnant and non-pregnant women (Dimova et al., 2011), the composition of this pool changes dynamically throughout pregnancy (Zhao et al., 2007). For example, in pregnant women, the fraction of circulating Tregs that express relatively high levels of human leukocyte antigen (HLA)-DR is increased compared to non-pregnant women, and these Tregs are highly effective at suppressing effector T cells (Teffs; Schober et al., 2012). During parturition, especially if premature, the suppressive activity of peripheral blood Tregs decreases (Schober et al., 2012). Granulocytic myeloid-derived suppressor cells (MDSCs), which, like Tregs, can inhibit Teffs, increase in the peripheral blood of healthy pregnant women throughout all trimesters; whereas, the numbers of monocytic MDSCs remain unchanged (Kostlin et al., 2014). Conventional CD11c^+^ DCs, which play an essential role in inducing the response of antigenically naïve T cells and B cells to antigens, are present in the circulation in high numbers during the first trimester compared to healthy non-pregnant women, and decrease as pregnancy progresses with the lowest absolute numbers by the third trimester (Della Bella et al., 2011). The circulating CD11c^+^ DCs of the third trimester display a phenotype that suggests incomplete maturation, with relatively high levels of CD80, CD86, CD40, and CD83, but not HLA-DR; whereas, this cell type in non-pregnant women displays high levels of all of these proteins. The reduced expression of HLA-DR is associated with a decreased capacity of this CD11c^+^ DC population to allogeneically stimulate T cells, suggesting that this phenotypic alteration might be relevant to limiting maternal responses to the fetus (Della Bella et al., 2011). Given these observations, it is plausible that a combination of alterations in the phenotype and function of immune cells in the maternal circulation in combination with the selective trafficking of these cells to the decidua (Arck and Hecher, 2013) and to the feto-placental unit (Shechter et al., 2013; Jeanty et al., 2014) may play an important role in sustaining a healthy fetal allograft/maternal host relationship.

### Impact of Immunotolerance on the Fetus and Neonate

There is growing evidence that both the maternal and fetal immune systems contribute to a healthy allograft/host relationship by reciprocal immune alterations via feto-placental immune trafficking. This in turn affects the health of the developing fetus and the neonate. Mold et al. (2008) have elegantly shown that maternal hematopoietic cells are found in the fetus as part of normal pregnancy and contribute to the generation of fetal suppressive Tregs that limit responses to non-inherited maternal HLA haplotypes *in utero*. Although the immune system of the developing fetus has been largely inaccessible for study, studies on cord blood have provided some insights into this relationship.

Granulocytic MDSCs predominate in cord blood at birth (Rieber et al., 2013). MDSCs are crucial in expanding the Treg populations in a cell contact- and indoleamine 2,3-dioxygenase (IDO)-dependent manner (Zoso et al., 2014) in human cord blood. In fact, in humans and mice, IDO is expressed by tolerogenic DCs and results in the peripheral conversion of naïve CD4 T cells into Tregs. Despite the predominance of these highly suppressive cell types in cord blood, neonates may not be as highly immunosuppressed at birth as previously thought. For example, human fetal DCs isolated from lymph nodes can properly respond to *in vitro* stimuli, which supports this hypothesis (Mold et al., 2008). Therefore, active suppression by regulatory populations of potential neonatal effector cells may be utmost importance for a healthy pregnancy outcome.

### Impact of Immune Perturbations on Pregnancy Outcomes

Perturbations in the abovementioned innate and adaptive immune system components that occur in early and late gestation have been documented to cause pathologic pregnancy outcomes, such as fertility problems, defective placentation through abnormal spiral artery remodeling, miscarriages, abortions, fetal deaths, preterm labor, pre-eclampsia, and intrauterine growth restriction (IUGR). HO-1 is believed to be a major regulator of the innate and adaptive immune systems, and may be involved in the interaction between the two systems during pregnancy (Soares et al., 2009; Koliaraki and Kollias, 2011). Many pregnancy, fetal, and neonatal complications can be attributed to perturbations in the maternal and fetal/neonatal immune systems, and a deficiency in HO-1 may be one such predisposition that disturbs allograft/host immune homeostasis.

## Effect of HO-1 Deficiency on Pregnancy Outcomes

### Impact of Variability in HO-1 Expression

The expression of HO-1 is genetically variable in humans due to either polymorphisms in the number of (GT)n dinucleotide repeats in the HO-1 promoter region (with longer repeats being associated with a decrease in HO-1 gene expression) or HO-1 mutant alleles. Moreover, a relative deficiency in HO-1 has been linked to a number of pregnancy complications. In both humans and mice, it can cause a chronic inflammatory state with an increased susceptibility to oxidative injury; whereas, the complete absence of HO-1 is lethal. However, there are also conflicting reports on the role of HO-1 polymorphisms in pregnancy complications. Denschlag et al. (2004) have shown that idiopathic recurrent miscarriages are associated with shorter (GT)n dinucleotide repeat lengths (≤27); while longer repeat lengths (>25) have been reported to be linked to late onset and less severe forms of pre-eclampsia (Kaartokallio et al., 2014).

In our laboratory, we have observed that HO-1 is highly expressed in the placenta, and appears to play a role in angiogenesis, placental vascular development, and the regulation of vascular tone during pregnancy (Zhao et al., 2009; Wong et al., 2012). In addition, a maternal deficiency in HO-1 in mice was found to affect fetal growth and maternal-fetal hemodynamics during early to mid-gestation due to changes in placental angiogenesis and differentiation of uNK cells (Zhao et al., 2008, 2009, 2011; Wong et al., 2012). Others have also reported that a deficiency in HO-1 is associated with decreased uNK cells at fetal implantation sites and results in defective spiral artery remodeling along with aberrant expression of vascular factors, such as vascular endothelial growth factor (VEGF) and placental growth factor (PGF). This consequently leads to hypertension in pregnant mice, which is reversible by CO administration (Linzke et al., 2014).

In addition, because HO-1 can affect oocytes, ovulation, and the corpus luteum, its deficiency may adversely impact fertility. For example, HO-1-deficient mice produce fewer oocytes after hormonal stimulation despite having a normal numbers of follicles, a lower fertilization rate, a lower number of corpus lutea, and a higher rate of apoptosis in corpus lutea compared to wild-type (Wt) mice (Zenclussen et al., 2012).

These data do suggest a role for the HO/CO system in maintaining a healthy pregnancy, starting from the oocyte through the corpus luteum, fertilization, implantation, placentation, and beyond. In addition, changes in hormonal balance during pregnancy can in turn affect the HO/CO system and impact normal and pathological pregnancies (Acevedo and Ahmed, 1998; Zenclussen et al., 2014). Therefore, a detailed study of genotypes along with characterization of the immune cell populations in the various pregnancy disorders may provide insights into their pathophysiology and could lead to the development of novel diagnostic technologies and therapies involving modulation of the HO/CO axis.

### Impact of HO-1 Deficiency on Placentation

The placenta is a complex organ with metabolic, hormonal, and immune functions. The integrity of its vasculature is critical in maintaining these functions at the feto-placental interface. In murine pregnancies, day E10.5 corresponds to the end of the first trimester in the human pregnancy, and is an important gestational age (GA) when spiral artery remodeling occurs and the labyrinth (where feto-maternal exchange occurs) starts to develop. In pregnant HO-1 heterozygous (HO-1 Het) mice, placental HO-1 expression and total HO activity change as a function of GA. After E14.5, HO-1 is primarily found in the spongiotrophoblasts in the junction zone of the mouse placenta (Wong et al., 2012). At E16.5, there are significant vasculature differences in the labyrinth between Wt and HO-1 Het placentas due to differences in placental angiogenesis (Zhao et al., 2011). Although, basal placental HO activity is greatest at E15.5 for both pregnant Wt and HO-1 Het mice, HO-1 protein decreases in placentas at E15.5 in HO-1 Het dams, along with a concomitant increase in HO-2 compared to Wt placentas (Zhao et al., 2009). Total microvasculature vessel volumes in the labyrinth are largely decreased in the HO-1 Het placentas and independent of fetal genotype compared to Wt mice (Zhao et al., 2011). These vascular structural alterations in HO-1 deficient feto-placental units could render the mother and the fetus more susceptible to environmental stressors, weakening the feto-placental barrier and, thus, increasing immune trafficking and disturbing the allograft/host immune homeostasis.

### Effect of HO-1 on Immune Cells and Sustaining the Immunotolerance between the Allograft and Host

Heme oxygenase-1 is vital for the allograft/host immune homeostasis. The majority of knowledge on this topic arises from organ transplant studies. For example, tolerogenic DCs, which express high levels of IL-10 and low levels of pro-inflammatory cytokines, such as IL-12p70, have been shown to prolong allograft survival in an HO-1-dependent manner in a heart transplant model (Moreau et al., 2009). In addition, upregulation of granulocytic (CD11b^+^ Gr1^+^) MDSCs after a chronic exposure of mice to lipopolysaccharide (LPS) inhibits T-cell responses in an HO-1-dependent manner, which delays skin allograft rejection; whereas, inhibiting HO-1 with tin mesoporphyrin (SnMP) restores allogeneic-driven T-cell proliferation (De Wilde et al., 2009). Because the fetus can be considered an allograft, HO-1 also positively impacts pregnancy outcomes by maintaining a tolerogenic DC profile and increasing Tregs, hence sustaining feto-maternal immunotolerance. For example, inhibiting HO-1 by zinc protoporphyrin (ZnPP) decreases Tregs at the feto-maternal interface and results in fetal allograft rejection; whereas, upregulation of HO-1 by cobalt protoporphyrin (CoPP) maintains tolerogenic DCs and increases Tregs and prevents fetal allograft rejection (Sollwedel et al., 2005; Zenclussen et al., 2007; Schumacher et al., 2012).

**FIGURE 1:**
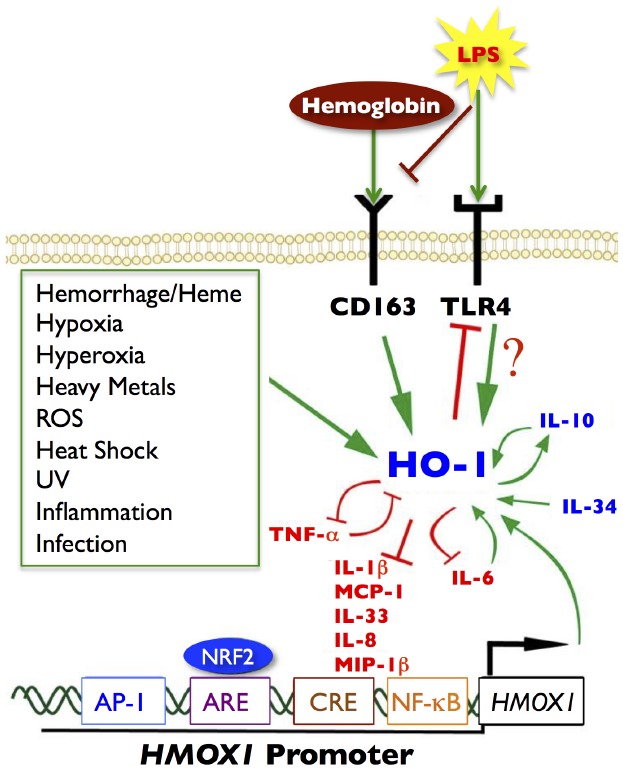
**Signaling pathways of HO-1.** Green arrows represent increased HO-1 expression or activity. Red blocked lines represent inhibition or decreased expression. Several noxious stimuli, highlighted in the green box, are known inducers of HO-1, as well as hemoglobin/heme through the CD163 receptor and LPS through the TLR4 receptor. Anti-inflammatory cytokines are represented in blue; pro-inflammatory cytokines are depicted in red. There are many cytokine signaling loops that involve HO-1 activity or expression, including a positive feedback loop between HO-1 and IL-10 (anti-inflammatory), and a negative feedback loop between HO-1 and TNF-α (pro-inflammatory). HO-1 can inhibit cytokine and chemokine responses such as IL-1β, IL-8, IL-33, MCP-1, and MIP-1β. The pro-inflammatory chemokine IL-6 (upregulates HO-1), which in turn inhibits IL-6 to limit inflammatory responses. Several transcription factors can also bind to the HO-1 promoter (*HMOX1*), notably NRF2 at the ARE site, but also AP-1, CREB, and NF-κB can bind to the promoter at independent binding sites and induce HO-1 expression. Modified and adapted from Ambegaokar and Kolson (2014) with permission from Bentham Science.

At the cellular level, HO-1 is constitutively expressed in tolerogenic DCs; however, its expression decreases during DC maturation *in vitro*. In addition, in human and animal immune cells (e.g., DCs, monocytes, and macrophages) HO-1 can also inhibit LPS-induced phenotypic maturation and inflammatory cytokine secretion and promote anti-inflammatory cytokine production (Figure 1). HO-1 is also constitutively expressed by CD4^+^CD25^+^ Tregs in human peripheral blood, and is inducible in CD4^+^CD25^–^ naïve T cells upon activation (Biburger et al., 2010). However, there is general acceptance that the suppressive functions of Tregs are dependent upon the HO-1 expression status of the DCs (George et al., 2008) rather than that of Tregs (Zelenay et al., 2007; Biburger et al., 2010). Interestingly, overexpression of HO-1 produces an anti-proliferative effect on immune cells, resulting in an increased number of cells remaining in the G0/G1 phase (Woo et al., 1998) suggesting a cell intrinsic regulatory mechanism.

Hence, the HO/CO system may provide some protection against pathological pregnancies by promoting the development of a maternal tolerogenic phenotype. In addition, we believe that such a protective role of HO-1 may also be at work in the fetus and neonate, enabling them to develop an immunotolerant phenotype, and hence a healthy allograft/host relationship. Because upregulation of the HO/CO system is associated with the inhibition of LPS-induced changes in immune cells, it is possible that such protective effects might also limit the effects of “pathological inflammation.”

### Impact of HO-1 Deficiency on the Immunomodulatory Functions of Angiogenic Factors

**FIGURE 2:**
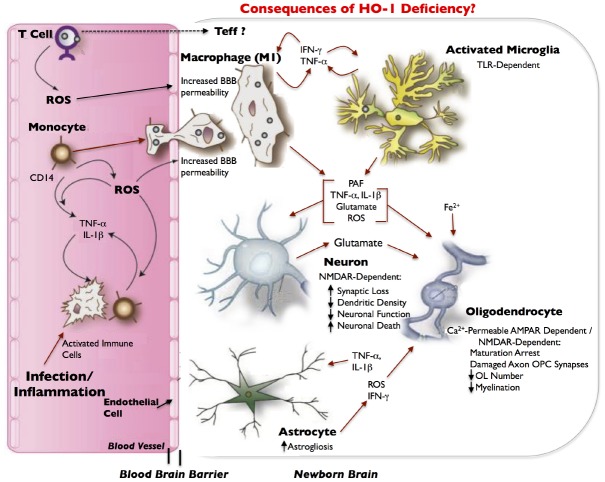
**Model of inflammatory/infection-mediated neuropathogenesis in HO-1 deficiency.** Infection/inflammation-activated immune cells, including CD4^+^ T cells and CD14^+^ monocytes, circulate through the blood and produce reactive oxygen species (ROS). Infected or activated immune cells can also release the pro-inflammatory cytokines, TNF-α and IL-1β, which ROS can exacerbate. ROS contribute to increased blood–brain barrier (BBB) permeability, which can allow for increased entry of activated immune cells (e.g., monocytes) from the blood into the newborn brain. Effector T-cells (Teffs) lymphocytes may also enter the brain. As monocytes enter the brain, they differentiate into macrophages, and release IFN-γ in addition to TNF-α and activate microglia. Both macrophages and microglia can produce IFN-γ and TNF-α in a positive feedback loop, and can release a host of neurotoxic and oligodendroglial toxic factors. Most neurotoxicity is initially limited to synaptic loss and decrease in dendritic density that is dependent on the NMDA-type glutamate receptor. This leads to eventual loss of neuronal function and finally neuronal death. We hypothesize that in HO-1 deficiency, an increase in TNF-α, IL-1β, IFN-γ, ROS, glutamate, and free iron (Fe^++^) results in the arrest of oligodendrocyte maturation, axon-oligodendrocyte synaptic damage, a decrease in oligodendrocyte numbers, a decrease in myelination and results in periventricular leukomalacia (PVL). Activated astrocytes have abnormal glutamate metabolism, leading to excess glutamate release and excitotoxicity. TNF-α and IL-1β stimulation of astrocytes can further increase glutamate release. Astrocytes, microglia and macrophages are potent inducers of HO-1, which is downregulated in infected macrophages and in infected brains, and thus may also contribute to neuropathogenesis of infection; neurons demonstrate very limited expression of HO-1. Red arrows indicate potential neurotoxins or direct neurotoxic/oligodendroglial toxic pathways. Modified and adapted from Ambegaokar and Kolson (2014) with permission from Bentham Science.

During normal placental development, HO-1 regulates angiogenic factors specifically at the decidua and mesometrial lymphoid aggregate of pregnancy (MLAp) regions of the placenta. HO-1 deficiency, on the other hand, alters the expressions of placenta angiogenic factors and results in development of a thinner and structurally-altered placenta in mice. Two of these angiogenic factors, hypoxia-induced factor-1α (Hif-1α) and hepatocyte growth factor (Hgf) have immunomodulatory functions (McGettrick and O’Neill, 2013; Imanirad et al., 2014; Molnarfi et al., 2014) and are decreased in HO-1-deficient placentas at E10.5 (Zhao et al., 2011). Normally, Hif-1α, which is abundant in inflammatory M1 macrophages but not in M2 macrophages (Takeda et al., 2010), attenuates Treg development and activates IL-17-producing Teff (Th17) cells, which promotes neutrophilic inflammation. Hgf stimulates tolerogenic DCs and Tregs, decreases Th17 cells, and downregulates markers of T-cell activation, thereby conferring immunotolerance (Benkhoucha et al., 2010; Molnarfi et al., 2013). Hif-1α and Hgf contribute to this important immunoregulatory balance not only in the placenta; but also, in the central nervous system (CNS; Benkhoucha et al., 2010; Dang et al., 2011). Therefore, a decreased expression of Hif-1α and Hgf, in the context of HO-1 deficiency, may disturb immune homeostasis at the feto-placental junction, and thus perturb fetal allograft tolerance, especially when challenged by environmental stressors, particularly those that result in increased immune cell trafficking and pathological inflammation. Moreover, because these two immunoregulatory angiogenic factors are also expressed in the CNS and Hif1-α specifically has a key role in angiogenesis in the CNS and myelination (Yuen et al., 2014), we hypothesize that in the HO-1-deficient fetus, these factors can adversely affect neurodevelopment and result in periventricular leukomalacia (PVL; Figure 2).

## Types of Pathological Pregnancies and the Protective Role of the HO/CO Pathway

*In vivo*, the HO/CO system and immunomodulation are closely linked through a multitude of complex pathways (Tullius et al., 2002; Soares et al., 2009). Its immunomodulatory role has been described in many conditions such as organ transplantation, rheumatologic diseases, multiple sclerosis, and ischemia-reperfusion injury, pulmonary fibrosis, pulmonary hypertension, diabetes, *in vitro* LPS-stimulated immune cells, and in the prevention of pathological pregnancies (Remy et al., 2009; Zenclussen et al., 2011; Amano and Camara, 2013). The bioactive byproducts of HO (CO and bilirubin) mediate their anti-inflammatory and cytoprotective actions principally by downregulating pro-inflammatory and upregulating anti-inflammatory responses (Koliaraki and Kollias, 2011). CO regulates various protein kinases, [e.g., p38 mitogen-activated protein kinase (p38MAPK)], phosphodiesterases, and ion channels, which causes vascular relaxation, and prevents apoptosis. CO has immunomodulatory effects on endothelial cells and neutrophils, promotes tolerogenic DCs and macrophages, and induces expansion of Tregs. In addition, CO inhibits generation of reactive oxygen species (ROS) in macrophages, and inhibits activation of Teffs, downregulates TLR4 expression, and inhibits the production of pro-inflammatory cytokines, such as IL-6 and IL-17. Collectively, these effects of CO promote immunotolerance and protect tissues from injury (Brouard et al., 2000; Soares et al., 2009; Burt et al., 2010; Amano and Camara, 2013; Wegiel et al., 2013). In addition, bilirubin is a free radical scavenger, which also inhibits human lymphocyte responses, and biliverdin can inhibit complement *in vitro* (Nakagami et al., 1993; Haga et al., 1996).

Hence, these effects of the HO/CO pathway may be important in maintaining a healthy allograft/host relationship, and CO may have a role as an immunomodulator in the prevention or treatment of pregnancy complications. Because CO is a gas, its beneficial effects could also be extended to the fetus and neonate.

### Infertility, Abortions, and Miscarriages

In women with infertility, failed *in vitro* fertilization (IVF) attempts, or recurrent spontaneous miscarriages, a decrease in Tregs, an increase Th17 cells, an imbalance of Th1/Th2 cells and their cytokines, as well as abnormalities in MDSC and NK-cell populations and function have been reported in peripheral blood and decidua (Saito et al., 2010; Dimova et al., 2011; Lash and Bulmer, 2011; Schlossberger et al., 2013; Kim et al., 2014; Kamoi et al., 2015; Zhao et al., 2015). Such immune imbalances, however, have not been observed in electively-induced abortions in humans (Caliendo, 2007; Zenclussen et al., 2007). Therefore, infertility and spontaneous miscarriages could be a function of such disturbed immune homeostasis, and careful modulation of the HO/CO pathway might help restore homeostasis and improve pregnancy outcomes.

The use of metalloporphyrins, synthetic analogs of heme, to modulate the HO/CO pathway has provided some insight into its relevance in pregnancy in murine abortion-models. For example, the administration of CoPP increases HO-1 and HO-2 expression significantly in spongiotrophoblasts, labyrinth, and giant cells, and prevents abortions by upregulating the cytoprotective, anti-apoptotic molecule Bag-1 and neuropilin-1, a marker for Tregs, in the placenta. In contrast, inhibiting HO with ZnPP increases abortions. This protection of HO from abortions is attributed to the immunoregulatory role of Tregs (Sollwedel et al., 2005). Moreover, CO administration, in addition to preventing abortions, improves litter weights in animal models (Zenclussen et al., 2011; El-Mousleh et al., 2012), suggesting a protective effect of HO-1 upregulation and increased CO production in the neonate.

Infection and inflammation probably disrupts this delicate immune balance, which may be accentuated in HO-1 deficiency. For example, Kahlo et al. (2013) have shown that after an *in vitro* LPS challenge, normal human trophoblasts harvested in the first trimester are able to upregulate HO-1; however, HO-1 upregulation is diminished in trophoblasts from spontaneous abortions, which is associated with a decreased expression of Bag-1 and fetal allograft rejection. Thus, individuals deficient in HO-1 may be at a higher risk for adverse pregnancy outcomes under stress (e.g., infection and inflammation). Gene-based therapy using adenoviral transfer of the HO-1 gene has been shown to prevent abortions in mice, but only if expression is not too high (Zenclussen et al., 2006). Therefore, interventions to upregulate the HO/CO pathway might be considered as a means of alleviating early and/or late pregnancy complications, although control of the appropriate dose will likely be important.

### Pre-eclampsia

Pre-eclampsia is diagnosed when there is an onset of hypertension beyond the 20th week of gestation as defined by the American College of Obstetricians and Gynecologists (ACOG) criteria (Schroeder and ACOG, 2002). Its pathogenesis is still being investigated, but is believed related to vascular, endothelial, and immunologic alterations. HO-1 can regulate placental angiogenesis through both VEGF and sFlt-1 (soluble VEGFR-1); sFlt-1 is produced in mouse and human placentas during later stages of gestation and is an important marker for pre-eclampsia with increased levels correlating with increasing severity of pre-eclampsia (Hirashima et al., 2003; Ahmed, 2011; Anderson et al., 2012). It has been suggested that the development of pre-eclampsia in HO-1 deficiency might be facilitated by increasing sFlt-1 secretion (Ahmed, 2011). In contrast, upregulation of HO-1 and its byproduct CO can inhibit sFlt-1 secretion, thereby, alleviating pre-eclampsia (Ahmed, 2011). Because LPS can trigger the release of sFlt-1 from monocytes (Redecha et al., 2009), perinatal infections might further complicate or worsen pregnancy outcomes, particularly in women who are prone to pre-eclampsia.

Immunologic alterations, such as generalized inflammation and activation of certain immune pathways, are thought to play important roles in the pathogenesis of pre-eclampsia. At a cellular level, pre-eclampsia is associated with a predominance of M1 inflammatory macrophages at the feto-placental interface, activation of granulocytes and monocytes, a decrease in iTregs, and abnormalities in uNK cells and Th17 cells (Cerdeira et al., 2012; Faas et al., 2014; Fu et al., 2014; Wallace et al., 2015). To date, the immunomodulatory effects of the HO/CO system on macrophages in pre-eclamptic placentas have not been well defined. In a spontaneously hypertensive rat model, induction of HO-1 by heme enhances immunomodulatory M2 macrophages and suppresses inflammatory M1 macrophages and their production of the pro-inflammatory chemokines MCP-1 and MIP1-α, which stimulate macrophage infiltration (Ndisang and Mishra, 2013). In contrast, M2 macrophages are believed to be beneficial and promote immunotolerance in pregnancy, but they have also been shown to release high levels of iron after tissue injury and hemorrhage (Sica and Mantovani, 2012). Therefore, we speculate that in some pregnancy complications, especially in HO-1-deficient humans, a predominance of M2 macrophage may lead to a greater tissue iron load and result in a “reversed” phenotype, where tissue injury may still occur due to an iron-associated oxidative injury.

Administration of exogenous CO to pregnant HO-1-deficient mice has been shown to directly induce uNK cell proliferation possibly through the modulation of interferon-gamma (IFN-γ) and prevent the development of a pre-eclampsia-like syndrome and IUGR, independent of sFlt-1 and sEng (Linzke et al., 2014). It has been shown that offspring born to pre-eclamptic mothers are at an increased risk developing metabolic syndrome later in life due to prenatal programming (Barker hypothesis) via epigenetic effects (Tenhola et al., 2003; Vatten et al., 2003). In a spontaneous hypertensive rat model, the induction of HO-1 decreases total cholesterol and triglyceride levels and improves glucose metabolism by potentiating insulin signaling, thereby decreasing the likelihood of developing metabolic syndrome (Ndisang and Mishra, 2013).

### Preterm Labor

The etiologies of preterm labor are still under debate, but development of a “pathologic” inflammation by the mother is probably an important contributing factor (Capece et al., 2014). The major role of immune system alterations in preterm labor can be best demonstrated by comparing it to normal parturition at term (Nagamatsu and Schust, 2010a). Vince et al. (1990) reported that in early and term human deciduas, almost half of the cells are leukocytes (CD45) and of the hematopoietic lineage, emphasizing the importance of the immune system in pregnancy. In normal parturition, a “physiologic” inflammation is initiated by monocytes and macrophages that infiltrate the term placenta, maternal decidua, and the fetal membranes and contribute to spontaneous membrane rupture and normal labor; whereas, the infiltration of monocytes and neutrophils confer post-partum decidual involution (Halgunset et al., 1994; Thomson et al., 1999; Shynlova et al., 2013a,b).

In preterm labor, this well-orchestrated immune sequence is lost. For example, neutrophils become the predominant decidual and myometrial leukocytes (Shynlova et al., 2013a). In human preterm deliveries, the tolerogenic immune phenotype switches to an inflammatory phenotype. Here, the suppressive capacity of Tregs decreases despite total Tregs remaining the same and selective immunotolerance for the fetus is lost, which subsequently results in preterm delivery or “allograft” rejection (Schober et al., 2012). In addition, a pathological activation of complement, neutrophils, monocytes, and Teffs have been observed in both human and mice preterm labor further supporting the theory of immune imbalance and allograft rejection (Gervasi et al., 2001; Diamond et al., 2007; Saito et al., 2010; Shynlova et al., 2013a; Gonzalez et al., 2014). Moreover, it has been shown that the preterm labor inducing effects of pathological complement activation can be eliminated by HO-1 induction (Gonzalez et al., 2014).

### Preterm Premature Rupture of Membranes, Fetal Inflammatory Response Syndrome, Funisitis, Chorioamnionitis, and Neurodevelopmental Outcomes

Preterm premature rupture of membrane (PPROM) is associated with inflammation in most cases and may be linked to collagen, matrix, hematologic, and coagulation abnormalities (Capece et al., 2014). In some instances of PPROM, a continuum of infection, fetal inflammatory response syndrome (FIRS), funisitis, and chorioamnionitis ensues and can lead to adverse neurodevelopmental outcomes (Burd et al., 2012). In a co-stimulation assay using peripheral blood mononuclear cells (PBMCs) from maternal/fetus pairs, Steinborn et al. (2005), have shown that maternal PBMCs, after stimulation with PBMCs derived from their respective fetuses, have an increased stimulation index if mothers had PPROM. This suggests that a disruption of feto-maternal tolerance occurs; whereas, in maternal/fetus pairs without PPROM, PBMC co-stimulation assays show decreased stimulation indices (Steinborn et al., 2005). The continuum of infection, FIRS, funisitis, and chorioamnionitis requires the activation of umbilical cord endothelial cells and the infiltration of maternal inflammatory cells across the placental layers toward the fetus. For example, FIRS is associated with maternal peripheral blood leukocytosis (Bartkeviciene et al., 2013), and a predominance of activated neutrophils and monocytes in the cord blood of newborns with funisitis (Kim et al., 2009). In contrast, in LPS-stimulated term umbilical cord blood neutrophils, bilirubin, a product of the HO reaction, induces antioxidants superoxide dismutase (SOD) and HO-1 expression and decreases the production of the inflammatory cytokine IL-8 and the chemokine MIP-1β in a dose-dependent manner, conferring protection from LPS (Weinberger et al., 2013). A role for HO-1 deficiency in the context of infection and protection by endogenous HO-1 induction is well defined in some neurodegenerative diseases (Ambegaokar and Kolson, 2014). In LPS-treated mice, HO-1 induction by statins inhibits complement and decreases apoptosis in fetal cortical neurons, thereby conferring protection from fetal cortical neuronal injury (Pedroni et al., 2014).

### HO-1 and Perinatal Infections

A number of human and mouse studies have shown the contribution of pregnancy-associated infections and inflammation to adverse pregnancy outcomes (septic abortions, PPROM, FIRS, preterm delivery, and chorioamnionitis) and fetal/neonatal outcomes (early onset sepsis, autism, learning dis-abilities, schizophrenia, abnormalities of neuronal migration, and PVL). Maternal bacterial infections during mid-to-late pregnancies are probably the most clinically relevant for causing such adverse neonatal outcomes. Among very low birth weight (VLBW) infants, gram-negative bacterial infections, especially *E. coli*, can cause increased morbidity and mortality. Dorresteijn et al. (2015) have recently shown that LPS stimulation selectively downregulates HO-1 expression in PBMCs, monocytes, granulocytes, macrophages, and DCs from healthy male donors via stimulating Bach1, which transcriptionally represses HO-1 expression. However, similar LPS stimulation results in upregulation of HO-1 expression in mouse lung macrophages and human monocytic leukemia cell lines (Dorresteijn et al., 2015). Therefore, further research is necessary to better characterize HO-1 responses in immune cells during a healthy normal pregnancy, as well as during gram-negative infections during pregnancy and in the neonate and associated complications.

Vertical transmission of maternal viral infections [e.g., herpes simplex virus (HSV), human immunodeficiency virus (HIV), and cytomegalovirus (CMV)] can adversely affect pregnancy, fetal, and neonatal outcomes. For example, HSV and HIV can cause encephalitis and/or neurodegeneration. A relative deficiency in HO-1 has been suggested to play a role for viral encephalitis (HSV, HIV) in non-pregnant individuals (Schachtele et al., 2012; Gill et al., 2014). On the other hand, HO-1 has been reported to have a protective role in HIV-induced neurocognitive disorders (Ambegaokar and Kolson, 2014). Thus, further studies are required on whether HO-1 deficiency adversely affects the offspring’s neurodevelopment during a maternal HSV or HIV infection. In addition, it has been recently shown that in an *in vitro* CMV model using human PBMCs, HO inhibition by SnMP resulted in an expansion of CD8 cytotoxic T-cells, which may be helpful in controlling this viral infection (Bunse et al., 2014); these effects were also found to be cell-type specific, as uNK cells and DCs were not affected.

Heme oxygenase-1 is found to be protective in septic abortions due to gram-positive (*Listeria monocytogenes*; Tachibana et al., 2011) and -negative (*Brucella abortus*; Tachibana et al., 2008) infections in murine models. A common mechanism is that both gram-positive and -negative bacteria may decrease HO-1 expression in trophoblastic giant cells and increase apoptosis leading to abortions, which can be prevented by the induction of HO-1. Trichomonas is an extracellular parasitic organism that is associated with preterm delivery in humans. In a murine model of trichomoniasis, this infection resulted in an increased incidence of septic abortions that were associated with a decreased in HO-1 expression in uterine tissues and a concomitant increase in Th17 responses as reflected by an increase in RORγt expression, a transcription factor that promotes Th17 differentiation (Woudwyk et al., 2012).

## Possible Pharmacological Therapies Affecting HO-1 Expression and/or Immune System in Pregnancy

### Statins

These compounds are 3-hydroxy-3-methyl-glutaryl–coenzyme A (HMG-CoA) reductase inhibitors, which are known for their use in reducing cholesterol levels. They have been shown to have anti-oxidant, anti-inflammatory, and immunomodulatory properties (Muchova et al., 2007). We have shown that statins can induce HO-1 expression in a tissue-specific and also in a statin-specific manner (Hsu et al., 2006). For example, atorvastatin increases HO-1 expression in the heart, and both rosuvastatin and atorvastatin increase HO activity, increase tissue CO content and bilirubin in the heart, and increase plasma bilirubin, effects that collectively confer protection from oxidative stress (Hsu et al., 2006; Muchova et al., 2007). Simvastatin can induce HO-1 in human umbilical vein endothelial cells *in vitro* (Hinkelmann et al., 2010). Pravastatin has been shown to have the most promise for use in the prevention of pre-eclampsia since it does not cross the placenta and exerts its anti-inflammatory and immunomodulatory effects by suppressing sFlt and sEng. A recent study by McDonnold et al. (2014) demonstrated that the adverse consequences of pre-eclampsia in offspring, such as the late manifesting metabolic syndrome, can be alleviated by pravastatin. Statins have been shown to modulate the immune system by upregulating HO-1 mRNA expression in murine macrophage cultures (Chen et al., 2006). In pre-eclampsia, increased inflammation does not precede, but rather follows its onset since vascular imbalances seem to be important in the pathogenesis (Ramma and Ahmed, 2011; Faas et al., 2014). This suggests that the anti-inflammatory effects of the HO/CO pathway might be important in ameliorating pre-eclampsia after it is diagnosed clinically. However, statins are not FDA-approved for use in pregnancy. Because pravastatin is hydrophilic and does not cross the feto-placental barrier, it was chosen as the drug of choice for the “*Statins to Ameliorate Early Onset Pre-eclampsia Trial*” (Ramma and Ahmed, 2014). Furthermore, the National Institutes of Health (NIH) started a pravastatin pharmacokinetics trial in pregnant women at high-risk for pre-eclampsia (Costantine et al., 2013). However, the results of these two clinical trials are not currently available.

### Aspirin

Acetyl-salicylic acid (aspirin), an irreversible cyclo-oxygenase inhibitor, is anti-pyretic, analgesic, and anti-inflammatory. Low-dose aspirin has been proven effective for highly morbid Low-dose aspirin is also utilized for the pre-conceptional treatment of infertility, and for the post-conceptional prevention of recurrent miscarriages in some women (Lambers et al., 2009; Dentali et al., 2012; de Jong et al., 2014). However, its utility in the prevention of pregnancy complications has been under debate. In a recent multicenter, double blind, randomized control clinical trial [“*Effects of Aspirin in Gestation and Reproduction (EAGer)*”], pre-conception low-dose aspirin use as a primary prevention strategy did not increase live birth rates nor prevent miscarriages if the women had a prior history of miscarriages (Schisterman et al., 2014). Low-dose aspirin, also has not been effective in prevention of pre-eclampsia (Cantu et al., 2015). It has been shown that aspirin pre-treatment, increases HO-1 activity and protein expression *in vitro* in human umbilical cord endothelial cell cultures and decreases oxidative injury and increases survival of endothelial cells (Grosser et al., 2003). However, therapeutic doses of aspirin failed to induce HO activity and HO-1 protein levels *in vivo* when given to healthy human subjects in conjunction with simvastatin or α-lipoic acid (Bharucha et al., 2014). Further studies are needed in pregnancy.

### Immunomodulatory Therapies

Most animal studies have shown that an activation of the innate immune system and subsequent release of detrimental cytokines in the placenta and fetus occur within several hours of encountering an inflammatory stimulus. Most studies using animal models have been designed as “pre-treatment” strategies of pregnant dams using a tumor necrosis factor (TNF)-α-suppressing drug, such as pentoxifylline (Gendron et al., 1990), or a TNF-α receptor agonist, such as etanercept (Carpentier et al., 2011). Despite being proven effective as preventative measures in rodent pregnancies, these compounds cannot be recommended for use in humans during pregnancy, although there is evidence that pentoxifylline may increase pregnancy rates in sub-fertile women as reported in a recent Cochrane review (Showell et al., 2013).

### Cell-Based Immunotherapies

In a recent study using intradermal desensitization with paternal or third party peripheral lymphocytes for recurrent spontaneous abortion patients, Tregs increased in peripheral blood while Th17 cells decreased. A favorable cytokine profile was achieved in a majority of these women, who then proceeded to have a healthy pregnancy after immunotherapy (Wu et al., 2014). Whether these cell-based immunotherapies could be used for infection-related pregnancy complications remains to be proven. Although data are lacking for pregnant women, studies in immunosuppressed post-transplantation patients have shown that adoptive transfer of viral-specific cytotoxic CD8 T lymphocytes can help control CMV infections. Targeting CMV and other viral, bacterial, and fungal infections during pregnancy may be possible utilizing cell-based immunotherapies. Mesenchymal stromal cells (MSCs) have immunosuppressive properties partly attributed to the HO-1 pathway and are used in graft vs host disease (GVHD) as a therapeutic strategy by some investigators (Stagg and Galipeau, 2013). In contrast, the involvement of HO-1 in MSCs immunosuppression has been recently disputed by a study by Patel et al. (2015) utilizing MSCs from healthy donor bone marrows and stimulating them with inflammatory mediators in an *in vitro* model. The use of MSCs in pregnancy cannot be advocated at this time.

## Conclusion

Infertility, miscarriages, abortions, pre-eclampsia, preterm labor, PPROM, infection/inflammation during pregnancy, maternal and fetal deaths, prematurity, small-for-gestational-age newborns, and resulting adverse neonatal neurodevelopmental outcomes are some of many the consequences of pathologic pregnancies. The causal pathways shared by these adverse outcomes may relate to the disrupted allograft/host immune homeostasis, which can be altered further in the context of a relative deficiency in HO-1. As with most protective mechanisms in the body, the HO/CO pathway can be influenced by host factors, such as HO-1 polymorphisms or challenged by environmental stressors, such as infection or inflammation. These environmental stressors result in “pathological” inflammation, perhaps more so in mother/fetus pairs unable to upregulate the HO-1 sufficiently to meet the demands.

Heme oxygenase-1 appears to have diverse protective roles in pregnancy, including in sustaining a healthy oocyte and subsequent fertilization, optimizing placental vascular development and spiral artery remodeling, facilitating a tolerogenic allograft/host environment, and extending these immunoprotective effects to the fetus, and subsequently to the neonate. The use of compounds or treatment strategies to upregulate this pathway in an immune cell-specific manner could be a promising approach to preventing, alleviating, and/or treating pregnancy complications, as well as adverse neonatal outcomes in the future.

### Conflict of Interest Statement

The authors declare that the research was conducted in the absence of any commercial or financial relationships that could be construed as a potential conflict of interest.
